# Mapping of novel loci involved in lung and colon tumor susceptibility by the use of genetically selected mouse strains

**DOI:** 10.1038/s41435-021-00159-z

**Published:** 2021-12-29

**Authors:** Andrea Borrego, José Ricardo Jensen, Wafa Hanna Koury Cabrera, Solange Massa, Orlando Garcia Ribeiro, Nancy Starobinas, Marcelo De Franco, Silas Fernandes Eto, Giacomo Manenti, Tommaso Antonio Dragani, Olga Martinez Ibañez

**Affiliations:** 1 Laboratory of Immunogenetics, Instituto Butantan, São Paulo, Brazil; 2 Diagnostic section, Instituto Pasteur, São Paulo, Brazil; 3 Laboratory of Development and Innovation, Instituto Butantan, São Paulo, Brazil; 4 Genetic Epidemiology and Pharmacogenomics Unit Fondazione IRCCS, Istituto Nazionale dei Tumori di Milano, Milan, Italy

**Keywords:** Immunogenetics, Inflammasome, Innate immunity

## Abstract

Two non-inbred mouse lines, phenotypically selected for maximal (AIRmin) and minimal (AIRmax) acute inflammatory response, show differential susceptibility/resistance to the development of several chemically-induced tumor types. An intercross pedigree of these mice was generated and treated with the chemical carcinogen dimethylhydrazine, which induces lung and intestinal tumors. Genome wide high-density genotyping with the Restriction Site-Associated DNA genotyping (2B-RAD) technique was used to map genetic loci modulating individual genetic susceptibility to both lung and intestinal cancer. Our results evidence new common quantitative trait loci (QTL) for those phenotypes and provide an improved understanding of the relationship between genomic variation and individual genetic predisposition to tumorigenesis in different organs.

## Introduction

This study aimed to map chromosomal regions involved in the regulation of susceptibility/resistance of mice in developing colon and lung tumors, induced by treatment with chemical carcinogen. AIRmax and AIRmin mouse strains, produced by bidirectional phenotypic selection on the basis of a high (AIRmax) or low (AIRmin) acute inflammatory response (AIR) were used as experimental model [1]. The selection process to obtain the two lines started from a population consisting of the balanced crossing of 8 inbred strains. The mixture of strains of different origins ensured a wide genetic variability in this population, thus bringing the model closer to the heterogeneity found in human populations. In addition, the progenitor strains have divergent sensitivities to colon and lung tumorigenesis reviewed in [2, 3] and thus genetic components related to organ-specific carcinogenesis were present in the background of the foundation population. Dimethylhydrazine (DMH) and its metabolite Azoximethane (AOM) are pro-carcinogens with tropism to colon and induce the appearance of tumors molecularly similar to non-familial colon cancers in humans. Previous mapping studies using several inbred susceptible and resistant mouse lines and crosses, as well as recombinant congenic strains (CcS/Dem), identified about 20 regions distributed across the chromosomes containing candidate modifier genes of colon carcinogenesis. These QTL are named *Scc* (susceptibility to colon cancer) and their large number is evidence of the polygenic nature of susceptibility/resistance to this type of cancer [4, 5]. Numerous mapping studies in humans have demonstrated the existence of at least 20 genomic regions and candidate genes in non-family colon cancer [6]. AIRmax and AIRmin lines are widely divergent in susceptibility to the development of tumors induced by chemical carcinogenesis regardless of the carcinogen employed and the affected organs [7–12]. AIRmin are resistant and AIRmax mice are very sensitive in developing colon tumors caused by DMH while under the same treatment, multiple lung tumors appeared only in the AIRmin mice [11]. There are no reports of induction of lung tumors by DMH in laboratory mice and it should be noted that even in the most sensitive strains to develop colon tumors, no invasive tumors and metastasis appear. The susceptibility of AIRmax and AIRmin mice to develop tumors in the colon and lungs after treatment with the same carcinogen (DMH) makes the model suitable for the research of common genes involved in their regulation. For this we produced, an (AIRmax x AIRmin) F2 population and treated all F2 animals with (DMH). At the end of the experiment we recorded the incidence, number, and size of colon and lung tumors. High-density genotyping was carried out for all individuals with the 2B- RAD technique (Restriction Site-Associated DNA genotyping) [13–15], to map the genetic loci that modulate individual susceptibility to colon and lung cancer.

## Results

### Colon and lung carcinogenesis

**Fig. 1:**
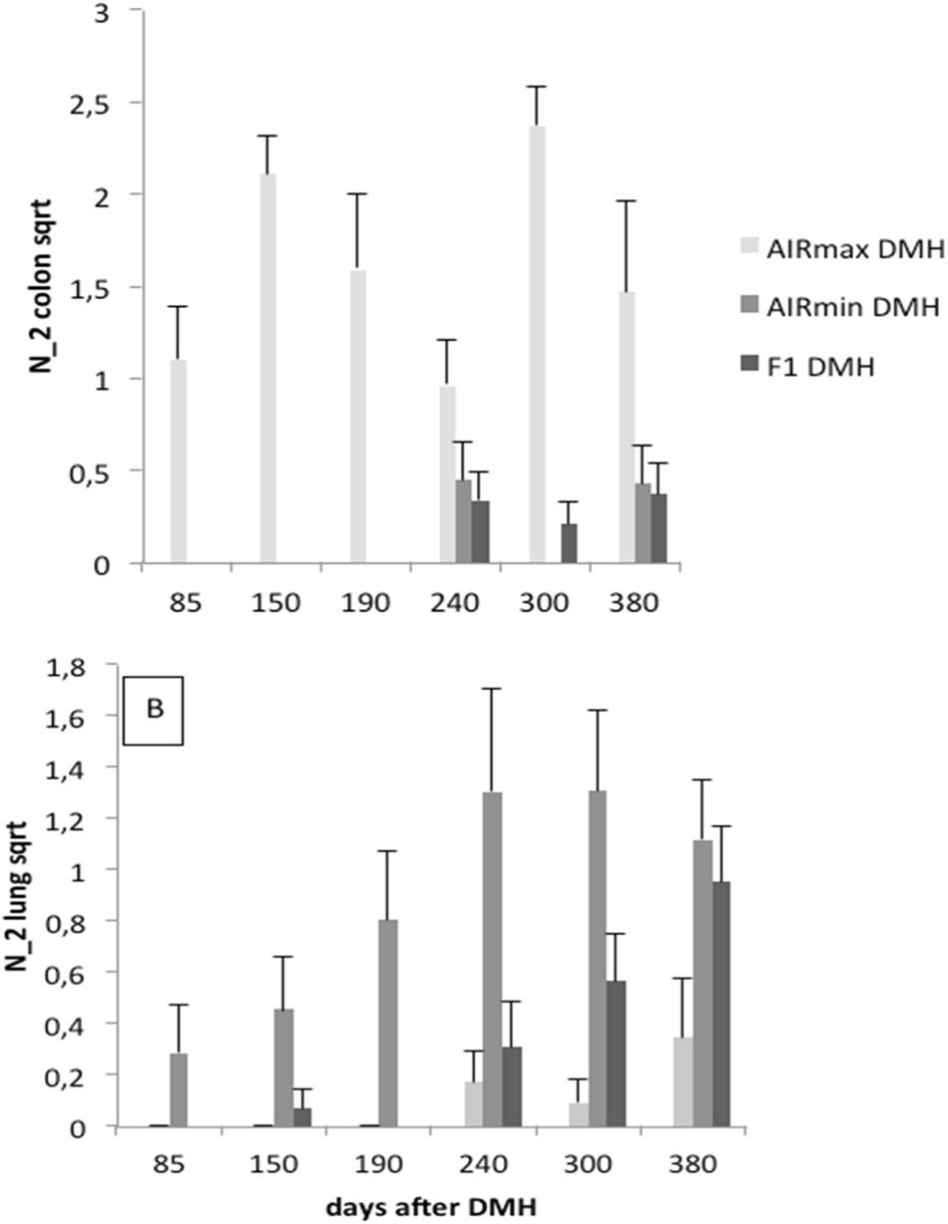
Development of DMH induced lung and colon tumors. AIRmax, AIRmin, and AIRmax × AIRmin F1 hybrids were injected ip with DMH (185 mg/kg. Colon (A) and lung (B) tumors were analyzed at different times after treatment. N2_colon sqrt = number of colon tumors with diameter >2 mm (square-root transformed values). N2_lung sqrt = number of lung tumors with diameter >2 mm (square root transformed values). Mean values and SD of 8–10 animals per group.

The phenotypic characterization was performed in the pedigree consisting of AIRmax, AIRmin grandparents (*n* = 22), (AIRmax x AIRmin)F1 parents (n = 20), and (AIRmax x AIRmin)F2 (*n* = 180) animals to evaluate the association between the transmitted alleles and the phenotypes presented by the individuals. The onset of colon and lung tumors in AIRmax, AIRmin and (AIRmax x AIRmin)F1 (Fig. 1) showed a clear inverse susceptibility between the two strains, confirming previous results [11]. Resistance to colon carcinogenesis is dominant in (AIRmax x AIRmin)F1 animals, similar to what occurs in crosses between inbred strains. On the other hand, the appearance of lung tumors was dominant in this group, in disagreement with the conditions of codominance found among inbred strains [16].

Table 1 shows the tumor incidence in the (AIRmax x AIRmin)F2 population at 300 days after treatment with the carcinogen. The frequencies of mice bearing colon or lung tumors were similar (28 and 25%, respectively), whereas about 18% of mice had both colon and lung tumors.

**Table 1:** DMH-induced colon and lung tumor incidence in AIRmax × AIRmin)F2 mice.

Negative	Colon	Lung	Colon + Lung
50	50	44	32
28.4%	28.4%	25%	18%

**Fig. 2:**
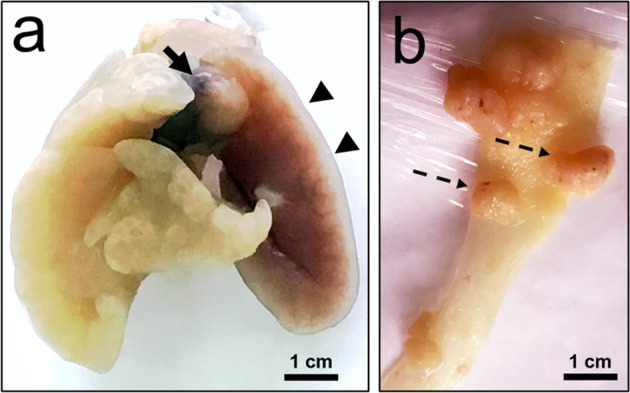
Macroscopic examination of DMH-induced lung and colon-rectal tumors from (AIRmax x AIRmin)F2 mice. **a** spherical mass (arrow) growing at the apex and between the right (arrowhead) and left lung lobes. **b** Pediculate (dotted arrow) and sessile polyps distributed in the colon-rectal epithelium.

**Fig. 3:**
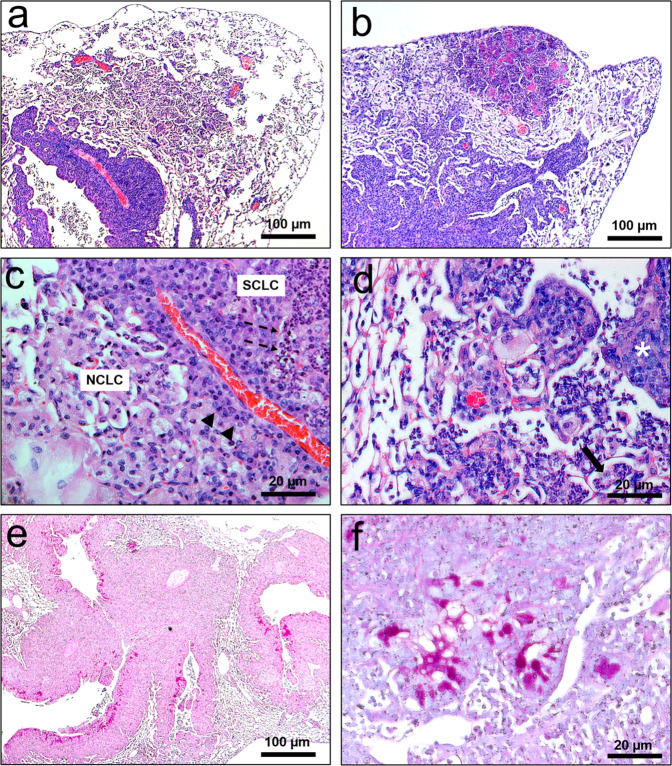
Histology of DMH induced lung tumor from (AIRmax x AIRmin)F2 mouse. **A** and **B** Shows, at lower magnification, multiple in situ lesions arising in the large airways and some small invasive carcinomas characterizing the bronchial-alveolar progression of lung tumors. **C** Greater resolution of tumor cellularity showing large, pleomorphic cells with abundant eosinophilic cytoplasm containing some vacuoles suggestive of small cell lung carcinoma (SCLC) combined with focal areas of non-small cell lung carcinoma (NSCLC) and poorly differentiated consisting mainly of cells with degenerative changes in the nuclei and cytoplasm (arrowhead) and in the periarteriolar area a modern plasmacytic leukocyte infiltrate. **D** shows tumor cells progressing to alveoli forming neoplastic mats and tumor nodule composed of cells arranged in clusters and nodular aggregates (asterisk) and an intra-alveolar neoplastic embolus (arrow). **E**, **F** mucin production (magenta-colored substance) in smaller and larger increases. H&E and PAS; Bar 20–100.

**Fig. 4:**
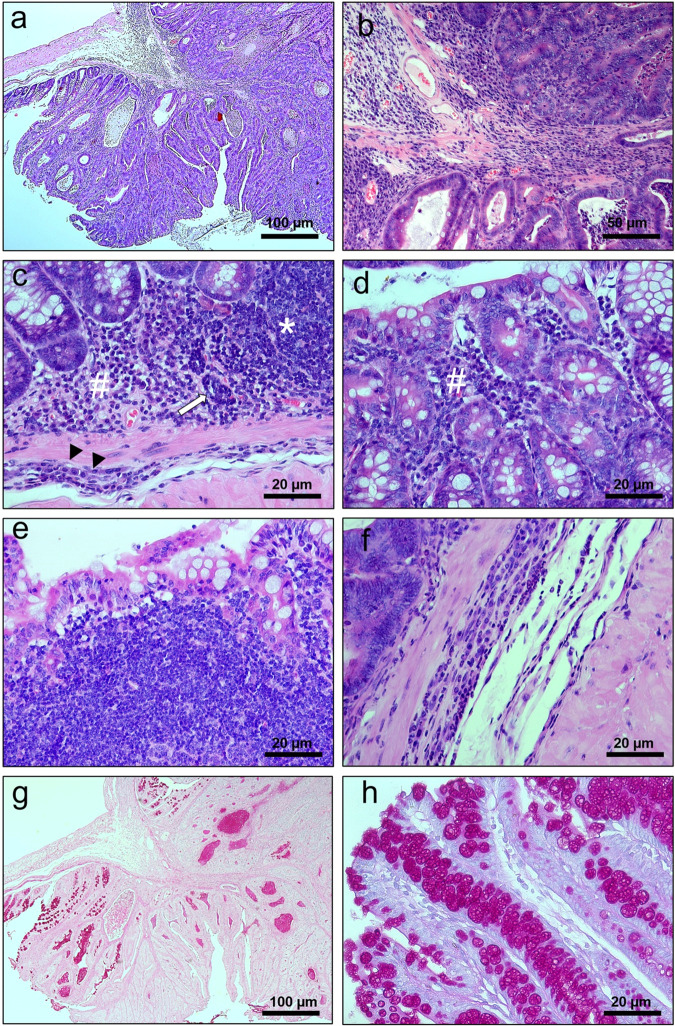
Histology of DMH induced colon-rectal tumor from (AIRmax x AIRmin)F2 mouse. **A**, **B** Pediculated polyp in smaller and larger magnification showing well-differentiated carcinoma, with level 4 invasion, invading the submucosa of the intestinal wall, without compromising the muscle layer classified by the Haggitt Classification System. This example shows a complex growth pattern indicating high-grade dysplasia shown in detail in **C** (*) leukocyte infiltrate, (#) desmoplastic reaction, and (arrow) presence of neoplastic emboli in the small vessel lumen of the pedicle submucosa. **D**, **E** progression of the leukocyte and neoplastic infiltrate from the base to the apex of the villi. **F** shows in greater resolution the invasion of the submucosa by neoplastic and leukocyte cell mats with dysplasia and vacuolization with a deposit of amorphous substance at the apex of the villi. **G**, **H** increased mucin production (magenta colored substance) shown in polyp and surrounding villi H&E and PAS; Bar 20–100.

Figures 2–4 shows the macroscopic aspect and microscopic features of DMH-induced colon and lung tumors from (AIRmax x AIRmin)F2 mice at 280 days after treatment.

Histopathological diagnosis: The pulmonary tumor model (Fig. 2a) gave rise to central tumors resulting from invasive lesions originating in the large bronchi, although occasional tumors have arisen in the peripheral lung of the bronchioles airways or alveolar ducts confirmed by microscopy analysis. In the pulmonary histopathologic analysis (Fig. 3), pre-invasive lesions consisted of cells with similar morphology to the invasive component and were considered carcinoma in situ lesions. Tumors occupy up to 70% of the lung volume, with peribronchial and perivascular invasion. The histopathological diagnosis of colorectal polyp (Fig. 2b) shown and described in Fig. 4 is suggestive of colon-rectal carcinoma arising from a malignant adenomatous polyp. Although PAS staining appears, tumors cannot be considered mucinous because staining occurs in less than 50% of the tumor area.

### Linkage analysis in (AIRmax x AIRmin) F2 mice

**Fig. 5:**
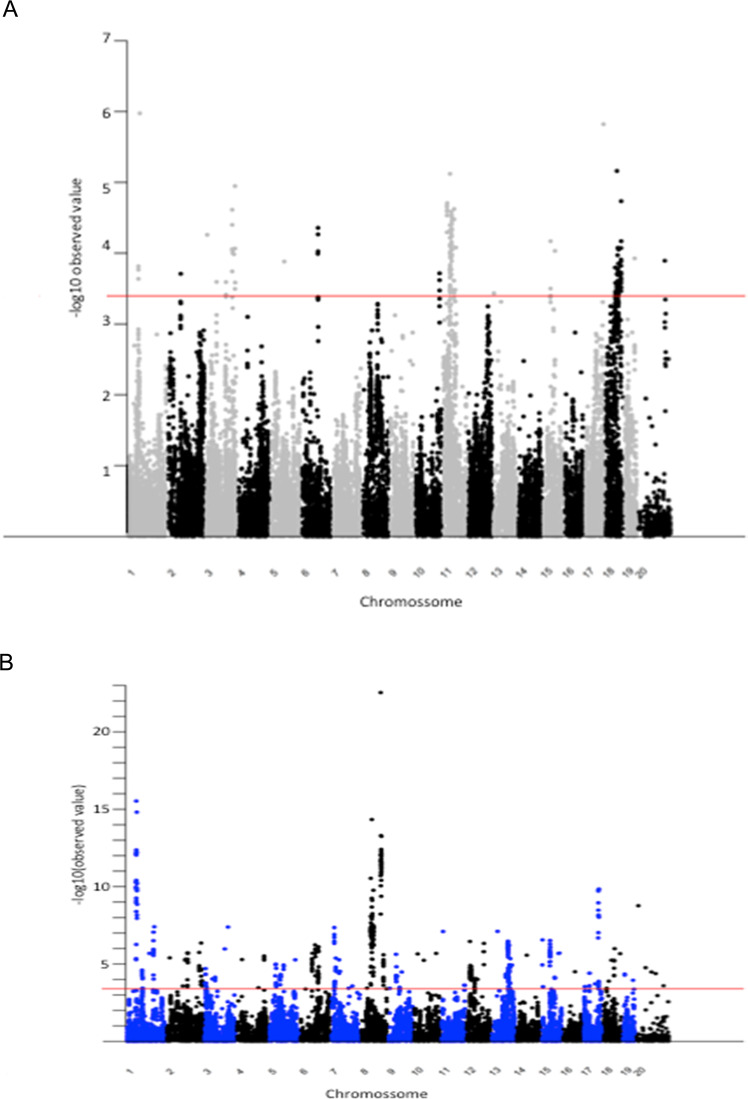
Genomic wide association analysis of DMH-induced colon (**A**) and lung (**B**) tumor multiplicity in (AIRmax x AIRmin) F2 mice. Manhattan plot of observed *P*-values in −log scale for 31,913 SNPs. The horizontal line indicates the genome-wide thresholds of significant association for colon and lung tumor phenotypes.

**Fig. 6:**
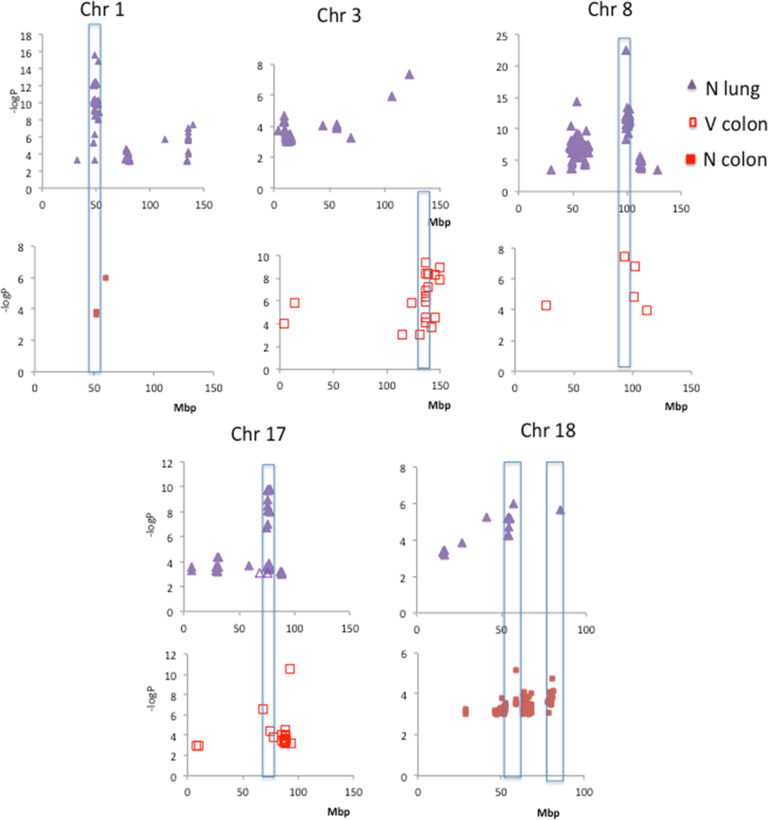
Details of the genomic screening for various phenotypes in some chromosomes: N lung = number of lung tumors; N colon = number of colon tumors; V colon = total volume of colon tumors. The regions with higher significance of association and with similar positions for two phenotypes were highlighted considering an approximate 10 Mb interval.

A total of 31913 SNPs were used for linkage mapping after quality control filtering. Parameters considered for tumorigenesis were the total number of tumors in each organ, the total number of tumors with mean diameter > 2 mm, and the total volume, by the sum of the volume of the tumors, calculated by the formula 4/3ΠR^3^. Thus, we have values to assess the incidence, multiplicity, and tumor development. Representative results are presented in Fig. 5. We observed clear signs of association with tumorigenesis phenotypes throughout the genome with several SNPs reaching statistical significance. In Fig. 6 we present the coordinates of regions containing SNPs showing the most significant association in some chromosomes. In addition to the specific regions for each phenotype, we observed coincident regions modulating colon and lung tumors, as shown in the distal portion of chromosomes 8, 17, and chromosome 18 (Table 2, Figs. 5 and 6).

**Table 2:** Candidate genes or QTLs for DMH-induced colon and lung tumors mapped by Genomic Wide Association analysis in (AIRmax x AIRmin) F2 mice.

			Observed *P*-values (−logP)			
Chrom	Position Mbp	QTL /candidate genes	N colon	V colon	N lung	V lung
**1**	44–52	*Stat1, Stat4*	3.81		**15.53**	
	58–59	*Scc20, Casp8*	**5.97**			
	134–139	*Inava*			**7.40**	
**2**	78–81	*Scc2, Sluc31*		**5.94**		**6.07**
	104–107	*Scc10, Sluc31, CD59a,CD59b*			**5.72**	
	174.535614				**6.35**	
**3**	12 a 13			**5.83**	3.40	
	104–106		3.59		**5.97**	
	122.535935				**7.38**	
	135–136	*Ccs3*	4.40	**9.36**		
	149–150	*Scc7*	4.95	**8.90**		
**4**	108–109	*Podn, Rab3b, Sluc43*		**6.43**		3.46
**5**	131–132	CpG island, TSS region			**5.27**	
**6**	19.5.32672				**5.57**	
	56–61			**5.47**	**5.12**	
	75–77				**6.23**	
	80–83	CD207 antigen Langerin	4.27	**7.79**		
	90–96	Sluc45			**6.11**	**14.68**
**7**	16	*C5ar1*			**7.36**	
	23.354340				**5.38**	
	40.561633				**5.27**	
	143–145	*Fadd Fas (TNFRSF6), Sluc8*		**7.32**	3.01	
**8**	54	*VEGF-C*			**14.33**	
	92–94	*Ccl17, Ccl22, Mmp2, MT1*		**7.48**		
	98.926798				**22.53**	
	101–102			**6.82**	**13.25**	
	111–112	*IL34*		3.95	**5.60**	
**9**	9 a 10	TSS region		**12.06**	3.43	3.23
**10**	40.675751				**5.62**	
	125–126			**5.21**		
**11**	37.859414		**5.12**			
**12**	17–18	*Odc1*			**6,45**	
	24–28			**5.19**	4.84	3.95
	87–88	*Ccs1*			**6.33**	
**13**	3.601751	*Tasor2*	3.44	**11.04**		
	29–30	CpG island, TSS region		3.48	**7.10**	
	39–39		3.31	**6.32**	3.26	
	82–85				**6.47**	
	95–95			3.46	**5.35**	
**14**	32.749958			**14.77**		
	106–107			3.00		5.35
**15**	57.53680				**6.55**	
	42–43	*Lfnq3* (lung function QTL3)			**6.52**	
**16**	53			**8.34**		3.76
**17**	67–68			**6.64**		3.13
	71–75	*Alk, Xdh, Lpin2*		4.43	**9.79**	3.08
	77–79	*Cyp1b1, Scc4, Sluc32*		3.78	**9.84**	
	92	CpG island, TSS region	**5.82**	**10.58**		
**18**	30.424859	*Mom3*		4.70		
	43	*Mcc*		4.84		3.47
	52–53	*Scc5*	3.46	**6.01**	**5.20**	
	56–58	*Sluc41*	3.54	**5.87**	**5.99**	
	63	*Apcdd1, Csfr1, IL17b*	4.07	**5.62**		
	84–90	*Cd226*		**5,67**	**5,67**	
**19**	10.721753				4.28	**5.85**
	24–25					**8.28**
	27.492760					**9.59**
**X**	15501131				**8.77**	

Ncolon = colon tumor muliplicity; Vcolon = total volume of colon tumors; Nlung = lung tumor multiplicity; Vlung = total volume of lung tumors.

Highly significant associations (−logP > 5 values) are highlighted in bold.

## Discussion

This study aimed to map genetic risk factors for lung and colon DMH-induced carcinogenesis. The AIRmax and AIRmin mouse lines are particularly powerful for this type of research since they differ widely in resistance/susceptibility to development of lung and colon tumors. The highly controlled conditions of the experimental design (breeding, treatment, animal facilities condition) minimize the influence of environmental variables, thereby reducing the confounding effects of gene-environmental interactions, a challenge for studies in human populations. High-density genotyping across the entire genome of the intercrossed F2 population of AIRmax/AIRmin animals using the 2B-RAD technique allowed for the high-resolution mapping of loci that modulate susceptibility to lung and intestinal cancer. DMH is a pro-carcinogen that is metabolized in the liver, producing reactive intermediates that are excreted through bile into the intestine or fall into the blood circulation; the ultimate metabolite methyl diazonium binds covalently to DNA, producing the pro-mutagenic O6 methyl guanine (O6Meg) lesion that induces GC to AC transitions in epithelial cells. These alterations induce activating mutations in oncogenes such as *Kras*, which are found in initial aberrant crypt foci lesions and in colon cancers, as well as in lung tumors in the mouse. Mutated *Kras* forms play a dominant role in driving metabolic reprogramming in several cancers [12, 17].

Tumor multiplicity in (AIRmax x AIRmin)F1 hybrids revealed dominance of resistance to DMH-induced colon cancer, and dominance of susceptibility to lung cancer. Nevertheless, in the (AIRmax x AIRmin)F2 population the incidence of colon or lung tumors was similar (28 and 25%). There was significant, albeit low, individual inverse correlation (*r* = −0.15 *p* = 0.045) in the F2 population between the incidence and progression of lung and colon tumors. Concordant susceptibility to both tumors was found in 18% of F2 mice, pointing to a link or identity in some genes controlling the development of both tumors. Histopathological analysis classified pulmonary tumors as carcinomas in situ and colon tumors as adenocarcinomas. Colon and lung derive from the foregut, the anterior part of the digestive tract; therefore some genes should regulate molecular pathways in both organs. However, lungs and digestive tract have different physiological functions and tissue-specific effects might play major influences in the group of genes acting in tumor onset and progression in each organ.

In the present genome screening, most SNPs that reached high statistical significance localize in non-coding regions of the genome, however, several significant SNPs are concentrated in specific regions of some chromosomes (Fig. 5). Nongenic cancer-risk SNPs are also usually found in human GWS studies [18, 19]. These genetic variants might affect the expression of nearby genes mapping to these regions, which might play a role in disease mechanisms in that tissue. Furthermore, we detected co-localization of chromosomal regions that control colon and lung tumor development. This may result from the presence of multiple closely linked genes, possibly controlling carcinogen processing or common carcinogenic pathways. Some of the several genes that physically map closely to the peak for association at these locus regions, highlighted in Fig. 6 and in Table 2, which are known to play roles in human cancers, are listed below.

For lung tumor multiplicity and progression phenotypes, the major association peaks were detected in chromosomes 1, 8, and 17. The region we mapped approximately at 50 Mb in chromosome 1, overlaps the region that spans the *Scc20* QTL which harbors the transcription factors *Stat1* and *Stat4* involved in the transcription of cytokines and angiogenic factors in lung tumors [20]. These mediators might also contribute to inflammation and colon tumor development, as observed by the significant association of SNPs mapping in this region with colon tumors; the region in chromosome 8 at 54 Mb harbors the *Vegf*-C (vascular endothelial growth factor C) gene which is the main regulator of angiogenesis in the process of tumor growth and metastasis. Some variants of this gene in humans have been associated with the risk of developing lung tumors [21, 22]. The chemokines genes *Ccl17* and *Ccl22* are localized close to the highest peak of association at 98 Mb. Ccl17 and Ccl22 are important for cell recruitment in inflammatory processes, including cells that infiltrate tumors, and the genes coding for metallothioneins (MT), a group of low molecular weight cysteine-rich proteins involved in protection against DNA damage, oxidative stress, and apoptosis are also located in that region. Increased expression of these genes correlated with development of some types of tumors including those of colon and lung in humans [23]. Significant linkage with colon tumor development was observed at this same chromosomal location; in Chromosome 17, the region near 74 Mb harbors the *Alk* gene (anaplastic lymphoma kinase) that has been targeted for the therapy of some types of lung cancer [24] and the *Xdh* (xanthine dehydrogenase) gene. Interestingly xanthine oxidase (XO), the oxidized form of xanthine dehydrogenase, forms uric acid and ROS that activate the NLRP3 inflammasome complex for the release of IL-1β, a central mediator in inflammatory processes [25]. Another neighboring gene, *Lpin2* (Lipin 2) is also involved in the activation of inflammasome by the purinergic receptor *P2rX7* activation pathway [26]. Accordingly, we found significant associations of SNPs mapping in this interval with colon tumors.

The highest significant associations with colon tumors localized at chromosomes 3, 8, and 18. The distal portion of chromosome 3, between 131 to 146 Mbp colocalizes with *Ccs3* (colon cancer susceptibility 3) QTL. This region harbors some candidate genes including *Sgms2* (Sphingomyelin synthase 2S), which has a role in inflammation-mediated tumorigenesis, such as colon cancer originating from colitis [27]; *Egf* (epidermal growth factor) whose product is secreted by tumor cells of the colon and contributes to the M2 polarization of macrophages associated with the tumor [28]; *NF-κB1* (nuclear factor-kappa B), that promotes cell proliferation, regulates immunological and inflammatory responses. Studies indicate that this gene is activated constitutively in malignant tumors and the action of some molecules with anti-tumor activity is attributed to the inactivation of this factor [29, 30]; a group of genes encoding alcohol dehydrogenases (*Adh1, Adh5, Adh6a, Adh6b, Adh7*) maps at 138 Mb. The activity of these enzymes which is dependent on polymorphisms, is implicated in the risk of colon cancer [31]. *Mcc* (mutated in colorectal cancers), localized at 44 Mb in chromosome 18 is involved in colon carcinogenesis. In colon cancer, the gene is silenced by methylation of the promoter, leading to a failure in repairing inflammation-induced DNA damage [32]. Decreased expression of this gene has been described in mouse lung tumors [33]. The region between 61 and 63 Mb contains *Apcdd1* (Adenomatosis polyposis coli down-regulated 1), which is regulated by the beta-catenin/TCF complex and its high expression contributes to the colorectal tumorigenesis [34], as well as *Il17b* (interleukin 17B), involved in lung tumors and metastases [35, 36] and *Csf1r* (colony-stimulating factor 1 receptor) that has been considered as a target in cancer therapy [37]. Finally, the region around 80 Mb harbors the gene encoding the CD226 receptor that regulates NK cells function, which is important in tumor immunity [38, 39]. On chromosomes 17 and 18, significant association was detected at previously described loci that regulate colon and lung cancer in the mouse, i.e., the QTLs Susceptibility to lung cancer 32 (*Sluc32*) and Susceptibility to colon cancer 4 (*Scc4*) on chromosome 17 (78 Mb) and *Sluc41* and *Scc5* on chromosome 18 (20–57 Mb). Our results are in agreement with previous publications, which indicate a subset of genes that are functionally or genetically related presenting pleiotropic effects in different organs [40].

Several studies have been carried out in humans, to quantify the degree of shared genetic basis between different cancer types. Large scale cancer genetic epidemiological consortia such as the Genetic Associations and Mechanisms in Oncology (GAME ON) and Genetic Epidemiology of Colorectal Cancer Consortium (GECCO) have conducted cross-cancer genomic analysis based on numerous publicly available GWAs datasets. Genome-wide genetic correlations have been evaluated between pairs of cancer types and in one of these studies, a correlation of 0.31 (*p* = 0.001) was found between lung and colorectal cancers, involving genetic variations in inflammation-associated components [41–45].

As a general summary, the present study suggests the participation of genes encoding transcription and angiogenic factors, DNA damage protectors, genes that participate in the activation of the inflammasome complex, as well as genes coding for mediators such as chemokines and interleukins that modulate the activity of immunocompetent cells on the resistance or susceptibility to the development of colon and lung tumors. Functional polymorphisms in these candidate genes or, more likely in their regulatory regions could have accumulated differentially in the AIRmax and AIRmin strains during the bidirectional selection process based on inflammatory reactivity.

The polygenic risk scores described in other GWAS studies have contributed to the genetic profiling of individuals with higher risk of non-familiar or sporadic cancers. The genome-scale study presented here supports the modeling of multigene panels for understanding the complex genetics of cross- lung and colon cancer individual risk, and indicates pathways for the study of therapeutic targets linked to inflammation control.

## Material and Methods

### Mice and treatments

AIRmax and AIRmin mice, F1 hybrids (AIRmax x AIRmin) F1 and the resulting F2 population of F1 x F1 intercross, (AIRmax x AIRmin) F2 were inoculated ip with 1,2-dimethylhydrazine (DMH) (Sigma Aldrich Chemicals) at a dose of 26.4 mg/Kg bw, beginning at 2 months of age and repeated weekly for another 6 weeks, totaling the dose of 185 mg/kg bw. Groups of AIRmax, AIRmin, and (AIRmax x AIRmin) F1 were sacrificed at various times after treatment, to verify the temporal evolution of the onset of tumors in internal organs, especially in the lungs and colon. The F2 animals were sacrificed 300 days after the last dose of the carcinogen. All experiments followed the national guidelines for the care and use of animals and were approved by the Committee for the Use of Animals (CEUA) of Instituto Butantan, São Paulo, Brazil, Protocol number: 6754030915

### DNA extraction

Tail tip DNA was extracted with E.Z.N.A. columns, according to the manufacturer’s instructions (Omega Biotech Inc. USA). Purity and concentration were determined in the Nanovue apparatus (GE) by the ratio of λ260/280 readings and integrity was analyzed in 1% agarose gel electrophoresis.

#### Restriction site-associated DNA genotyping (2B-RAD technique)

In this technique, only DNA fragments adjacent to sites of recognition of restriction endonucleases are sequenced. In our essay we used the enzyme CspCl, which has 283.329 cleavage sites in mice,(value obtained by in silico digestion through the genome). This enzyme recognizes an invariant sequence of 7 nucleotides, divided into 3 and 4 nucleotides separated by 5 variable nucleotides. It cleaves the genomic DNA upstream and downstream of the target site and, in total, CspCl produces 36 nucleotide-long fragments. All nucleotides within the DNA 36-mer fragments, but which are not part of cleavage sites, may vary and can be polymorphic. The 2B-RAD technique usually produces from 3 million to 5 million reads, whose distribution in all sites recognized by the enzyme is expected to yield 20–30X coverage to make the data reliable. The standard protocol consists of 6 phases: enzyme digestion, adaptor link, amplification, purification with beads, quantification, and sequencing. The 2B-RAD libraries were grouped and sequenced (single-end) in Illumina HiSeq 2500 apparatus. Sequencing data were analyzed using a custom computational pipeline (Bash script) and the following tools: Trimmomatic (trimming), BWA (alignment), Picard, Bamtools, GATK (haplotype caller). We used the CeGen-ISCIII Genotyping Service at Universidad de Compostela, Spain. Data are available upon request.

#### Statistical analysis of the association between genotypes and phenotypes

Statistical analysis and quality of genotyping data were performed with the PLINK program [46]. To verify the significance of the associations between genotypes and phenotypes, each SNP genotype received a code (0, 1, or 2) according to the number of the minor alleles present, in order to represent their additive effects in that variable. Associations with quantitative variables (multiplicity and volume of tumors) were tested with linear regression, considering variables of sex and family. Due to the large number of statistical tests, the correction for multiplicity was made by the Benjamini-Hochberg false discovery rate (FDR) method [47].

#### Histopathological examination

Fragments of lung and colon-rectal tissue were collected and fixed in 10% formalin buffer for 24 h. After fixation, the samples were dehydrated in a series of alcohol of ascending concentration (70%, 80%, 90%, and 100%), embedded in paraffin and sectioned blocks with a thickness of 5 µm, mounted on slides and stained with hematoxylin -eosin (H&E) and by special staining with Periodic Acid Schiff (PAS). The slides were blindly analyzed by a veterinary pathologist.
